# Civil Lawsuits as an Indicator of Adverse Outcomes in Healthcare

**DOI:** 10.3390/ijerph191710783

**Published:** 2022-08-30

**Authors:** Marcin Mikos, Jolanta Budzowska, Tomasz Banaś, Dorota Kiedik, Katarzyna Sygit, Elżbieta Cipora, Beata Karakiewicz, Mateusz Kaczmarski, Izabela Gąska, Olga Partyka, Monika Pajewska, Jakub Świtalski, Anna Badowska-Kozakiewicz, Andrzej Deptała, Anna Augustynowicz, Michał Waszkiewicz, Aleksandra Czerw

**Affiliations:** 1 Department of Economic and System Analyses, National Institute of Public Health—NIH—National Research Institute, 00-791 Warsaw, Poland; 2 Budzowska, Fiutowski & Partners. Attorneys-at-Law, 31-041 Krakow, Poland; 3 Department of Gynecology and Obstetrics, Jagiellonian University Medical College, 31-501 Cracow, Poland; 4 Department of Radiotherapy, Maria Sklodowska-Curie Institute-Oncology Centre, 31-115 Cracow, Poland; 5 Department of Population Health, Division of Public Health, Wroclaw Medical University, 50-345 Wroclaw, Poland; 6 Faculty of Health Sciences, Calisia University, 62-800 Kalisz, Poland; 7 Medical Institute, Jan Grodek State University in Sanok, 38-500 Sanok, Poland; 8 Subdepartment of Social Medicine and Public Health, Department of Social Medicine, Pomeranian Medical University in Szczecin, 71-210 Szczecin, Poland; 9 Department of Health Economics and Medical Law, Medical University of Warsaw, 01-445 Warsaw, Poland; 10 Department of Cancer Prevention, Medical University of Warsaw, 02-091 Warsaw, Poland; 11 School of Public Health, Centre of Postgraduate Medical Education of Warsaw, 01-826 Warsaw, Poland

**Keywords:** cost, healthcare system, burden, outcomes

## Abstract

The financial burden of adverse healthcare outcomes in Poland still remains unknown. The objective of the study was to estimate the cost of adverse healthcare outcomes in the Polish healthcare system. Cost calculation was performed on the basis of civil cases completed in Polish courts against doctors and healthcare entities. The research material consisted of 183 civil cases completed by a final judgment in 2011–2013. The case study was conducted in five out of forty-five district courts across the country. Out of 183 reviewed cases, 73 complaints ended up with favorable judgments (39.9%). The average value of the subject matter of the dispute was USD 78,675. The total expected value of lawsuits in the 183 reviewed cases was USD 11,299,020. The total amount awarded in 73 judgments from medical facilities to injured patients was USD 2,653,595, which on average means USD 36,351 per case. The average amount of awarded compensation was USD 33,317 per case. The average compensation amount in the analyzed cases was USD 11,724. The average one-time annuity for a patient was USD 11,788. The estimated costs of negative healthcare outcomes amounted to USD 8,000,000 per year.

## 1. Introduction

Adverse healthcare outcomes resulting in the harm of patients are a serious medical and economic burden for health systems. Patients receiving any healthcare service may experience adverse effects. An adverse outcome can be defined as “any suboptimal outcome experienced by the patient” [1]. One of the first studies assessing the range of the problem was the Harvard Medical Practice Study of 1984, where the incidence of medical injury was estimated at 3.7% of all hospital admissions in New York State [2]. Further analysis of data from the same region showed that the total economic costs of health expenses and reduced productivity associated with these events reached up to USD 878 million in 1989, USD 3.8 billion in 2008, and if extrapolated to the entire US, over USD 50 billion [3]. In a study published in 2008 by Van Den Bos et al., the value of the annual cost of events that resulted in the injuries of US patients was estimated at USD 17.1 billion [4].

Another study conducted later confirmed these values by determining the level of the total annual medical costs including loss of productivity in the US at USD 54.6 billion. It is worth mentioning that the legal awareness of patients is increasing, thus the number of claims against hospitals and healthcare professionals is increasing accordingly [5].

It is estimated that every 10th hospitalized patient in Europe experiences an adverse event or malpractice that could have been avoided [6,7]. In the United Kingdom, the burden of litigation costs on the health budget is between 15% and 20% of the total annual expenditure [8]. In other countries, the costs of treating the harms caused by adverse events reached almost 9.5% of total health expenditure [9]. Among 30 European countries, the value of annual costs of adverse events was estimated in the range of EUR 17–38 billion in 2015 [10].

In addition to the costs of further treatment and rehabilitation, adverse healthcare outcomes result in the need to compensate for actual harms caused to patients or their families, such as loss of earnings and worsening of the financial situation. Studies show that it also could cause an exacerbation of health inequalities since patients who experience those events have to stay longer in hospital or undergo further costly treatment and rehabilitation, which only exacerbates the inequality further [11,12,13]. Adverse outcomes are also a source of negative intangible sensations such as pain, suffering, stress, shame, stigma or separation from loved ones. They are also the subject of compensation; however, the amount of redress which is recognized by the court is based on a free assessment of the patient and/or on jurisprudence.

There are at least four groups of costs generated by adverse outcomes or malpractice: the cost of additional health care services necessary to fix any medical condition arising; the cost of prolonged absenteeism plus possible rehabilitation; disability pension; compensation for harm; costs of court proceedings and related costs; and punitive damages. In practice, the authors do not know any publication dealing with all the listed groups of costs [9].

In the Polish health care system, about 8.3 million people are hospitalized per year. Total annual health expenditures reach the amount of USD 35 billion, yet there is no registry or monitoring of adverse events on a national level. The cost of adverse events in the health system still remain unknown. The objective of the study was to estimate the cost incurred by the health system due to adverse medical events using data from civil law suits.

## 2. Materials and Methods

The financial burden of adverse outcomes in healthcare was calculated on the basis of civil cases completed in Polish courts against doctors and hospitals. The data sources were the files of 183 cases pending against hospitals in relation to claims for damages, compensation and pensions for damages caused during the treatment process and completed by a final judgment in 2011–2013. Due to the duration of the court proceedings, the actions that initiated the proceedings were filed in the years 2001–2013. The largest number of claims was filed in 2009 (43), and the lowest in 2001 (1). It is also worth mentioning that, in 2015, there was a reform contributing to difficulties in the functioning of the judicial system in Poland therefore analyzing court files after 2015 would not add more value to the study. The criteria for choosing cases were the following: subject of the case—the claim of the patient for medical error; defendant—hospitals; jurisdiction of the court—regional courts hearing cases over PLN 75,000 (Polish zloty—national currency was estimated to equal approximately USD 26,600); and deadline for the end of the proceedings—the proceedings had to be closed by a final court judgment without the possibility of further appeal. The primary methodological assumption of our study was that the judgment was issued in an impartial and independent manner. Based on the above, 183 court files pending in 5 district courts located in Kraków, Kielce, Łódź, Poznań and Tarnów were recovered. Pursuant to Art. 27 of the Code of Civil Procedure of the Republic of Poland, the court which is able to bring an action is the court of the place of residence or seat of the defendant. However, in the event that the jurisdiction of several courts is justified or an action is brought against several entities for which, according to the provisions of general jurisdiction, different courts have jurisdiction, the choice of court rests with the plaintiff. Table 1 presents the data in terms of population structure in those 5 districts. For comparison, total data for the district are presented as well [14]. It was found that the population structure in the analyzed districts did not differ from the population structure in Poland. Trials were conducted against the hospitals in respect of claims for damages, redress and disability pensions. The case study was conducted in 5 out of 45 district courts across the country (11.11%). All voivodships (units of the highest level of administrative division in Poland) were comparatively represented in the study. The courts from which the cases were included in the analysis covered a population of 7.6 million inhabitants, which is 19.67% of the Polish population.

The following elements of judgments were taken into account in the analysis of the acts:

1. Result of the proceedings.
2. The condition of the property of the plaintiff (financial condition).
3. The expected number of claims by patients.
4. The amount and type of benefits paid by hospitals.
5. The duration of the court proceedings.

## 3. Results

Out of 183 reviewed cases, 73 complaints ended up with favorable judgments (39.9%). The dominant group of injured claimants were patients in bad or very bad financial situations. In 126 cases (68.85%), the poor financial situation was the reason the injured patients were exempt from charges for the judicial costs. The exemption was available not only to a poor person (defined according to separate regulations) but also to a person whose payment of the judicial costs alone would damage himself and his family. This is court practice in the vast majority of cases. In the majority of cases (68.8%), plaintiffs were fully exempt from the court fees. The injured who were in a better financial situation were only partially relieved from the costs of the proceedings; namely, patients were not fully charged in 47 cases (25.68%), while 5.47% of the cases did not receive any exemption from the court fees.

The average value of the subject matter of the dispute was USD 78,675. The total expected value of lawsuits in the 183 reviewed cases was USD 11,299,020. Patients or their families most often applied in the main claim for amounts ranging from USD 49,020 to USD 98,040 (32.24% of cases). In 29.51% of cases, plaintiffs were claiming for less than USD 32,680. Amounts in excess of USD 326,797 very rarely occurred (2.19%).

In 60.1% of analyzed cases, hospitals did not incur any juridical costs. These were cases in which there were no grounds for recognizing the claims of the plaintiff, and consequently, no adverse medical event was identified. In 27.32% of cases, the defendant hospital was ordered to pay the costs of the trial.

In 37.70% of all cases, the most frequently awarded amount was the sum of redress which was the result of the proceedings. In 22.40% of cases, the court found that it was justified for the claim of the patient to be awarded for material damage suffered as a result of the adverse event. Provision of a monthly pension was granted in 13.11% of cases, while a capitalized pension (one-off) was awarded to 7.65% of plaintiffs.

The total amount awarded in 73 judgments from medical facilities to injured patients was USD 2,653,595, which, on average, means USD 36,351 per case. In all forms of court-ordered compensation for patients, the redress was awarded most frequently in all recognized claims. In 0.55% of all cases, redress was awarded in an amount of more than USD 163,399, while in almost 10% of all cases, the compensation exceeded the amount of USD 32,680. Remedies granted were mostly in the range of USD 24,510 (25.68%). The average amount of awarded compensation was USD 33,317 per case. The average compensation amount in the analyzed cases was USD 11,724. The average one-time disability pension for a patient was USD 11,788.

Among the 183 analyzed court cases, in 60% of files, defendants (hospitals) did not bear the expense of the trial in connection with the decision of the court that there were no grounds for recognizing the adverse event. In 30.05% of all cases, hospitals were charged for the costs of the trial. The amount of these costs in 9.84% of all cases exceeded the amount of USD 3268. The total cost of all cases reached USD 160,131. In the remaining cases, it was not possible to determine the court costs burden on hospitals. Even though hospitals in Poland have mandatory insurance in case any civil lawsuits occur, insurance companies may not cover the cost of the compensation and the costs of proceedings due to specific provisions of the contract or a narrow scope of insurance or a low insurance amount.

Adverse event suits are often associated with a high degree of complexity of the facts and above average difficulty of the evidence process, as estimated from the number of opinions issued in these cases. The total duration of the proceedings was determined from the date of the filing of the claim to the court until the date of the final judgment. It usually ranged between 18 and 24 months (13.66%). However, some of them lasted between 30 to 36 months (13.11%), while others lasted from 24 to 30 months (10.93%). The median duration of adverse event cases reached three years (39 months). It is noteworthy that the proceedings pending for less than 24 months ended unfavorably for the plaintiff in 83% of cases. On the other hand, the suits that lasted from 42 months to 84 months ended with a decision reflecting the demands of the plaintiff in 55% of cases.

The long duration of the proceedings generated additional costs in the form of the amount of interest for a lengthy trial, granted by the court. In almost every third case (31.15%), the defendant medical institution was obliged to pay interest on the amount of benefits granted to the plaintiff.

## 4. Discussion

As stated earlier, in terms of the subject of medical errors and civil lawsuits in Poland, there is no registry or monitoring of adverse events on a national level. The files of medical cases with several dozen volumes of files at the seat of these courts were assessed, and therefore, the method of examination also determined the limitation of the sample. Limitations of our study include the rather short period of observation (cases completed by a final judgment in 2011–2013) and the cost calculation method which is based on the compensation amount granted to patients. The four dimensions of outcome—claims, redress, pensions and duration—are considered separately in our study which could also be a considered as a limitation. Further studies are required to assess the total costs of adverse events in the Polish healthcare system.

Below, we have presented some similar studies conducted in other countries in this subject. However, those results cannot be directly compared due to different methodology, population, economic status of countries, healthcare system and period of analysis.

The value of claims over a three-year period, averaged over the five jurisdictions sampled, is USD 530,719. This value allows us to estimate (45 judicial districts) that claims paid to patients reach in total almost USD 8,000,000 per year for the whole country. It still appears to be much less compared to the US health system as David et al. estimated the cost of treatment and compensation to amount to USD 985 million in 2008 and USD 1 billion in 2009 [15]. Although it is worth mentioning that the number of positively settled claims is also higher among US patients, reaching 61% compared to 40% in the Polish population [16].

Stevenson et al. assessed whether the experience of being sued and incurring litigation costs affects the quality of care [17]. An inverse relationship between litigation costs and quality was found. However, as the authors emphasize, only a few of the 27 associations models were statistically significant, such as the doubling of indemnity payments was associated with a 1.1% increase in the number of deficiencies and a 2.2% increase in pressure ulcer rates.

The average amount of claims paid in one case in Poland was USD 36,351, which is significantly lower than in the US (USD 323,818), but on the other hand, it is close to Denmark, where it is USD 40,000. However, the amount granted in Poland is significantly higher than in Sweden (USD 20,000) and New Zealand (USD 4450) [18].

Polish patients, who most frequently claim for redress with lawsuits against hospitals and medical personnel for adverse events, belong mainly to the group of residents of large cities with low status. Court verdicts confirm the legitimacy of the claims of patients at a rate of 40%. It cannot be ruled out that adverse events also occurred in the patients in the other cases, but if they were not related to an injury, it was not possible to obtain compensation [19].

The expectations of patients in cases for damages incurred due to adverse healthcare outcomes outweigh the amounts judged by the courts. In the analyzed cases, the patients demanded a total of USD 11,299,020, while in court rulings, a total of USD 2,653,595 in compensation was paid to them, which amounts to 23.5% of the expected amount. The difference between the amount claimed and the amount awarded is due to the fact that the courts judging the entirety of the circumstances accompanying the adverse event do not link some of the ailments of the patients with the consequences of irregularities on the part of the medical staff. In the course of objectified proceedings, the courts also often reveal the circumstances on the part of the patient concealed in the claim (contribution to the damage/extent of the damage) and assess the actual extent of the damage differently.

The Allué et al. study evaluated the incidence and costs of adverse events registered in Spanish hospitals. Data were analyzed from medical records, adverse event records and related medical treatment costs from 12 hospitals in the years 2008–2010. [20]. Such events were observed in 7% of hospitalized patients, whose costs accounted for 16% of total medical costs. Incremental costs associated with these events increased from EUR 5 to 12,000 to the standard cost of treatment. In total, incremental costs accounted for EUR 88 million and 6.7% of the total cost of treatment. This value is the basis for estimating the potential savings in financing care while reducing the risk of adverse events in hospitals.

The Giraldo et al. study, in 2016, compared the rulings on adverse events in Spain and Massachusetts in the USA between 2002 and 2012 [21]. It was pointed out that adverse events in Spain were more likely to be of a high severity. This could have affected the fact that, in Spain, 98% of judgments resulted in compensation for the victims whereas patients were granted financial benefits in only 7% of the cases in the USA. The authors also used a Spanish database containing 270 rulings dealing with adverse events for their detailed qualitative analysis. The most common causes were diagnostic errors (25%) and surgical treatment (22%). In 32% of cases, the result was death of the patient. The average period between the occurrence of the event and the ruling was 8 years, while the average compensation level was approximately EUR 240,000 [22].

In Poland, court proceedings concerning adverse events last 3 years and 3 months on average, which is significantly longer than the average duration of civil court proceedings. Due to the duration of the proceedings and the resulting costs, court proceedings could be considered as a low-cost form of dispute resolution between medical institutions and affected patients. However, there is a need for out-of-court forms of resolution in the form of special committees adjudicating adverse events in administrative procedures, as well as mediation and amicable settlement of disputes between medical facilities and patients. Nonjudicial systems involving mediation and settlement proceedings might be considered as a more effective way of pursuing claims related to the occurrence of adverse outcomes in healthcare [23,24]. However, that system has only been operating in Poland since 2012. Pursuing claims is possible only in the event of a medical event that occurred after 1 January 2012 and as a consequence of health services provided in a hospital.

## 5. Conclusions

1. Estimated costs of adverse events in the healthcare system in Poland amounted to USD 8,000,000 per year, which represents 0.03% of the total annual health expenditure in Poland.
2. In 60% of cases, the court found no premises for the undesirable event. Adverse event court proceedings are highly contested and complicated.
3. Due to their long duration and costs, court proceedings are not the most effective and optimal way for a patient to obtain compensation for an injury caused by the occurrence of adverse events.

## Figures and Tables

**Table 1:** Population structure in terms of the number of adult women and men of working age and post-working age in the poviats of the cities of Tarnów, Łódź, Kielce, Poznań, in the Kraków district and in Poland in total in 2013.

		District Tarnów		District Łódź		District Kielce		District Poznań		District Kraków		Poland	
		*n*	%	*n*	%	*n*	%	*n*	%	*n*	%	*n*	%
Working age	men	38,175	39.1	232,536	37.1	67,279	38.7	182,897	38.4	93,813	42.1	12,859,401	40.8
	women	35,853	36.8	221,819	35.4	63,393	36.5	175,997	37.0	84,979	38.1	11,562,745	36.7
Post-working age	men	7271	7.5	47,958	7.6	13,447	7.7	35,310	7.4	14,206	6.4	2,181,191	6.9
	women	16,211	16.6	124,795	19.9	29,742	17.1	81,630	17.2	30,031	13.5	4,896,960	15.5
Total		97,510	100	627,108	100	173,861	100	475,834	100	223,029	100	31,500,297	100

*n*—number of people; %—percentage of the total.

## Data Availability

Not applicable.
